# Dynamic Changes in the Gut Microbiome at the Acute Stage of Ischemic Stroke in a Pig Model

**DOI:** 10.3389/fnins.2020.587986

**Published:** 2020-12-03

**Authors:** Julie Jeon, Jeferson Lourenco, Erin E. Kaiser, Elizabeth S. Waters, Kelly M. Scheulin, Xi Fang, Holly A. Kinder, Simon R. Platt, Michael J. Rothrock, Todd R. Callaway, Franklin D. West, Hea Jin Park

**Affiliations:** ^1^Department of Foods and Nutrition, College of Family and Consumer Sciences, University of Georgia, Athens, GA, United States; ^2^Department of Animal and Dairy Sciences, College of Agricultural and Environmental Sciences, University of Georgia, Athens, GA, United States; ^3^Regenerative Bioscience Center, University of Georgia, Athens, GA, United States; ^4^Neuroscience Program, Biomedical and Health Sciences Institute, University of Georgia, Athens, GA, United States; ^5^Department of Small Animal Medicine and Surgery, University of Georgia, Athens, GA, United States; ^6^Egg Safety and Quality Research Unit, U.S. National Poultry Research Center, USDA-ARS, Athens, GA, United States

**Keywords:** MCAO, swine model, microbial diversity, inflammation, acute stroke

## Abstract

Stroke is a major cause of death and long-term disability affecting seven million adults in the United States each year. Recently, it has been demonstrated that neurological diseases, associated pathology, and susceptibility changes correlated with changes in the gut microbiota. However, changes in the microbial community in stroke has not been well characterized. The acute stage of stroke is a critical period for assessing injury severity, therapeutic intervention, and clinical prognosis. We investigated the changes in the gut microbiota composition and diversity using a middle cerebral artery (MCA) occlusion ischemic stroke pig model. Ischemic stroke was induced by cauterization of the MCA in pigs. Blood samples were collected prestroke and 4 h, 12 h, 1 day, and 5 days poststroke to evaluate circulating proinflammatory cytokines. Fecal samples were collected prestroke and 1, 3, and 5 days poststroke to assess gut microbiome changes. Results showed elevated systemic inflammation with increased plasma levels of tumor necrosis factor alpha at 4 h and interleukin-6 at 12 h poststroke, relative to prestroke. Microbial diversity and evenness were reduced at 1 day poststroke compared to prestroke. Microbial diversity at 3 days poststroke was negatively correlated with lesion volume. Moreover, beta-diversity analysis revealed trending overall differences over time, with the most significant changes in microbial patterns observed between prestroke and 3 days poststroke. Abundance of the Proteobacteria was significantly increased, while Firmicutes decreased at 3 days poststroke, compared to prestroke populations. Abundance of the lactic acid bacteria *Lactobacillus* was reduced at 3 days poststroke. By day 5, the microbial pattern returned to similar values as prestroke, suggesting the plasticity of gut microbiome in an acute period of stroke in a pig model. These findings provide a basis for characterizing gut microbial changes during the acute stage of stroke, which can be used to assess stroke pathology and the potential development of therapeutic targets.

## Introduction

An estimated seven million adults in the United States suffer from stroke each year, making it the fifth leading cause of death and the first leading cause of long-term disability (Benjamin et al., 2019). The immune response and inflammation are major stroke components effecting severity, as they can significantly exacerbate the primary stroke injury and cause further cell death in the brain (Deb et al., 2010; Borgens and Liu-Snyder, 2012). High levels of systemic inflammation are closely associated with poor stroke outcomes in stroke animal models and patients (Di Napoli et al., 2001; Audebert Heinrich et al., 2004; Elkind et al., 2004; McColl et al., 2007, 2008). Interestingly, it has recently been demonstrated that the gut microbiome changes in response to stroke (Swidsinski et al., 2012; Yin et al., 2015; Durgan et al., 2019) and that modulating the gut microbiome can alter the poststroke inflammatory response, leading to improved recovery in rodent models (Benakis et al., 2016; Singh et al., 2016; Yamashiro et al., 2017; Spychala et al., 2018). Few studies have assessed the changes in the microbial populations during the acute stage of stroke, making it critically important to better characterize these microbial alterations to identify potential biomarkers for injury severity, recovery, and therapeutic targets.

It has been demonstrated that adjustments in the gut microbiome influence ischemic brain injury by altering immune homeostasis (Benakis et al., 2016; Singh et al., 2016) and neuroprotective cytokine production (Benakis et al., 2016). This suggests that the gut microbiome is another potential therapeutic target for stroke (Benakis et al., 2016; Singh et al., 2016, 2018; Winek et al., 2016; Benakis et al., 2020). Studies of gut microbiome changes in stroke have demonstrated decreases in both commensal and beneficial genera, increases in pathogenic genera in human patients (Swidsinski et al., 2012; Yin et al., 2015), and substantial changes in the phylum Firmicutes, Bacteroidetes, and Actinobacteria in stroke mice (Singh et al., 2016). Imbalances of the intestinal microbiota can lead to gut barrier dysfunction and impairment of stroke outcomes. In a mouse middle cerebral artery occlusion (MCAO) ischemic stroke model, the stroke mouse exhibited an imbalance in microbial communities, resulting in a reduction in intestinal motility and increased protein leakage in the gut (Singh et al., 2016). These changes correlated with increased brain invasion of proinflammatory T cells from the gut and significantly increased brain infarction (Benakis et al., 2016). These findings in a rodent stroke model indicate that the gut microbiome is drastically affected by stroke and plays a pivotal role in stroke severity. However, recent failures to translate findings in rodent stroke models have led to the desire to study stroke pathophysiology and therapeutic targets in more translational large animal models such as the pig (Fisher et al., 2009; Saver et al., 2009; Albers et al., 2011).

Pigs are a robust translational animal model for biomedical research, especially gut and brain research, due to the myriad of similarities to humans in physiology, anatomy, pathology, and eating behavior (Watanabe et al., 2001; Lind et al., 2007; Swindle et al., 2012; Kaiser and West, 2020). They are omnivorous and have similar intestinal size and length in proportion to humans, contributing comparable transit time to humans (Clouard et al., 2012; Heinritz et al., 2013). The gut bacterial diversity of pigs are similar to that of humans showing higher richness and lower evenness than other animals (Lamendella et al., 2011), and 96% of the functional genes found in the human gut metagenome was present in the pig gut metagenome (Xiao et al., 2016), suggesting that the complexity of the pig microbiota is comparable to that of humans. Moreover, pigs have similar brain size, gray and white matter composition, and cytoarchitecture having a gyrencephalic brain like humans and unlike rodents (Baker et al., 2017). These similarities in the brain are predicted to lead to more human-like stroke pathology in the brain and brain–gut interactions. Due to gut homology between humans and pigs, a number of experiments have been conducted in pigs to study the interplay between the gut microbiome and the immune system (Foster et al., 2003; Scharek et al., 2005; Wilson et al., 2005; Zhang et al., 2013), yet never in a stroke pig.

In the current study, we investigated the changes in gut microbial diversity and composition in a MCAO stroke pig model developed by our research team (Duberstein et al., 2014; Platt et al., 2014; Baker et al., 2017). The results of this study conducted in a translational large animal model will help characterize patterns of bacterial changes during the acute stage of stroke, potentially providing future insight into stroke severity, recovery, and therapeutic targets.

## Materials and Methods

### Stroke Induction and Confirmation Utilizing Magnetic Resonance Imaging

All experimental procedures were approved by the University of Georgia Institutional Animal Care and Use Committee, and the study was conducted in accordance with the recommendations of the NIH’s guide for the Use and Care of Laboratory Animals (AUP approval number: A2017 07-019-Y2-A16). Seven castrated male Landrace pigs (5–6 months old, 48–56 kg) were individually housed in a room in which the temperature was kept at 27°C, with a 12-h light/dark cycle.

Ischemic stroke was induced in pigs by middle cerebral artery occlusion (MCAO) as previously described (Duberstein et al., 2014; Platt et al., 2014; Baker et al., 2017). Briefly, pigs were administered Excede [5 mg/kg intramuscularly and fentanyl patch (100 mcg/h transdermally)] 1 day prior to the stroke surgery to prevent infections and to manage pain. Midazolam (0.2 mg/kg) and xylazine (2 mg/kg) were administered intramuscularly for presurgery analgesia and sedation. For anesthesia, propofol was injected intravenously, and prophylactic lidocaine (1.0 ml of 2% lidocaine) was administered locally to the laryngeal folds to facilitate intubation. Anesthesia was maintained with 1.5% isoflurane in oxygen.

A curvilinear incision began from the superior right orbit and extended to the rostral aspect of the auricle. The temporalis muscle was retracted, and a craniectomy was performed at the exposed local dura mater. The middle cerebral artery located at the distal part of the Circle of Willis was permanently occluded using a bipolar electrocautery forceps. After postoperative recovery, pigs were returned to their respective pens and monitored every 4 h. To reduce postoperative pain and fever, Banamine (2.2 mg/kg intramuscularly) was administered every 12 h for the first 24 h and every 24 h for the following 3 days poststroke.

Magnetic resonance imaging (MRI) was conducted 1 day poststroke using a General Electric 3.0 T MRI system to confirm ischemic stroke. Pigs were anesthetized using the aforementioned anesthesia protocol and placed in a supine position using an 8-channel torso coil. T2 fluid-attenuated inversion recovery (T2-FLAIR) and diffusion-weighted imaging (DWI) sequences were used in conjunction with apparent diffusion coefficient (ADC) maps to confirm the presence of ischemic lesions.

### Blood Collection and Proinflammatory Cytokine Analysis

Peripheral blood was collected prestroke and 4 h, 12 h, 1 day, and 5 days poststroke, and plasma was separated and stored at −80°C. Circulating tumor necrosis factor alpha (TNF-α) and interleukin-6 (IL-6) were quantified by ELISAs (R&D systems, Minneapolis, MN, United States) to determine changes in inflammatory response.

### Fecal Collection and Microbial DNA Extraction

Fecal samples were collected prestroke and 1, 3, and 5 days poststroke. Samples were obtained directly from the rectum using sterilized plastic fecal loops (5 cm within the rectum). To prevent any contamination during fecal collection, all materials were sterilized prior to sample collection. Pig anus was stimulated with a sterilized loop for defecation, and the stool was collected into a sterilized sample tube without any contact to the floor or body. The fecal samples were immediately frozen on dry ice and stored at −80°C until further analysis.

Bacterial DNA was extracted from fecal samples using a previously validated approach described by Rothrock Jr., Hiett et al. (2014). In this method, 330 mg of fecal material is subjected to a combination of mechanical and enzymatic processes using a modified version of the FastDNA Spin Kit for Feces (MP Biomedicals, Solon, OH, United States) and the QIAamp DNA Stool Mini Kit (QIAGEN, Valencia, CA, United States). DNA purification was carried out using the DNA Stool–Human Stool–Pathogen Detection Protocol of the QIAcube Robotic Workstation. Following purification, DNA concentrations were determined spectrophotometrically (Synergy H4 Hybrid Multimode Microplate Reader; BioTek, Winooski, VT, United States).

### 16S rRNA Gene Sequencing and Analysis

The extracted DNA samples were sent to the Georgia Genomics and Bioinformatics Core^1^ to sequence the 16s ribosomal RNA (rRNA) gene. The V3–V4 region was amplified using the S-D-Bact-0341-b-S-17 (5′-CCTACGGGNGGCWGCAG-3′) and S-D-Bact-0785-a-A-21 (5′-GACTACHVGGGTATCTAATCC-3′) primer pair (Klindworth et al., 2013). Samples were sequenced using an Illumina MiSeq platform (Illumina, San Diego, CA, United States). Sequencing data were provided as FASTQ files, which were merged and converted into FASTA files, and further analyzed using the QIIME pipeline v1.9.1 (Caporaso et al., 2010). Sequences were clustered as operational taxonomic units (OTUs) at 97% similarity, and representative sequences were aligned to the Greengenes database (gg_13_8_otus). Singleton OTUs and OTUs whose representative sequences could not be aligned were excluded from the analysis. The computed alpha-diversity indexes are as follows: number of observed OTUs, Shannon index, and evenness. Beta-diversity was computed using the weighted UniFrac distance matrix. This metric was chosen because it accounts for the phylogenetic relationship when calculating beta-diversity.

### Statistical Analysis

Data were analyzed using GraphPad Prism (Version 8.1.1; GraphPad Software, Inc., San Diego, CA, United States) and are shown as mean ± SEM. Paired *t*-tests were used to measure the contrasts between pre- and poststroke time points. Differences in beta-diversity were accessed by two-sample *t*-tests between each individual time point, and the non-parametric *P*-values generated by the Bonferroni’s multiple comparisons test were used for inferences. Regression analysis was performed to evaluate associations between microbiome changes and stroke severity. For all statistical tests, *P* ≤ 0.05 were considered significant, and trends were declared when 0.05 < *P* < 0.10.

## Results

### Magnetic Resonance Imaging Confirmed Ischemic Lesions 1 Day Poststroke

**FIGURE 1:**
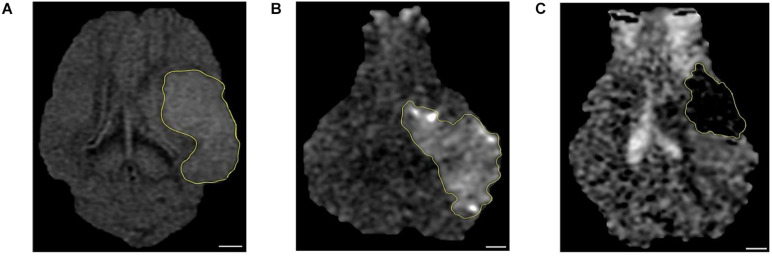
Magnetic resonance imaging (MRI) confirmed ischemic stroke in pigs. The hyperintense regions seen in **(A)** T2 fluid-attenuated inversion recovery (T2-FLAIR) and **(B)** diffusion-weighted imaging (DWI) sequences corresponded to **(C)** apparent diffusion coefficient (ADC) map hypointense regions thus confirming the presence of ischemic infarction 1 day post-middle cerebral artery occlusion (post-MCAO) surgery (*n* = 7). A scale bar is provided in each picture (6 mm), and the lesion area was outlined by yellow line.

Non-invasive MRI allows for real-time, longitudinal assessment of stroke pathophysiology and is a critical clinical tool commonly used to differentiate between stroke type and severity (González, 2012; Baker et al., 2017). We confirmed ischemic stroke 1 day poststroke in all animals in the study. Both T2-FLAIR and DWI sequences showed edematous lesions with bright hyperintense signal (Figures 1A,B), whereas ADC maps exhibited cytotoxic edema with dark hypointense regions due to restricted water diffusion (Figure 1C). Lesion volume, midline shift, and hemorrhage volume of the same cohort of pigs have been recently published (Kaiser et al., 2020). In brief, DWI sequences showed territorial hyperintense lesions at 1 day poststroke with an average volume of 9.9 ± 1.4 cm^3^. Analysis of T2-weighted (T2W) sequences revealed a significant (*P* < 0.01) increase in ipsilateral hemisphere volume indicative of cerebral swelling when compared to the contralateral hemisphere (26.0 ± 1.8 cm^3^ vs. 22.5 ± 1.4 cm^3^, respectively) and an associated midline shift of 2.5 ± 0.6 mm. Acute intracerebral hemorrhage was observed via T2Star sequences with a mean hemorrhage volume of 1.7 ± 0.1 cm^3^. Collectively, MRI results demonstrated that MCAO led to tissue-level damage including ischemic infarction, hemispheric swelling, pronounced midline shift, and intracerebral hemorrhage (Kaiser et al., 2020).

### Circulating TNF-α and IL-6 Levels Were Increased During the Acute Stage of Stroke in a MCAO Pig Model

**FIGURE 2:**
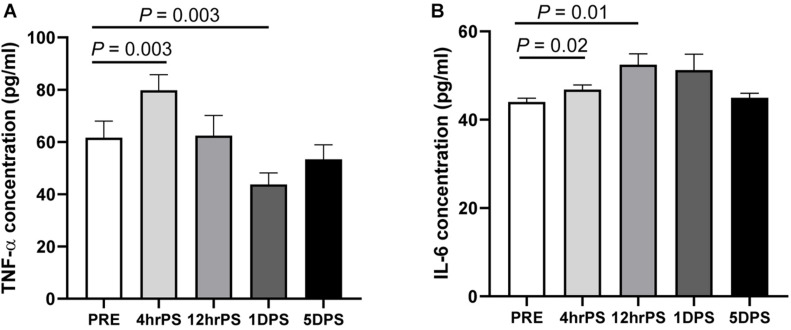
Plasma concentrations of proinflammatory cytokines were increased during the acute stage of ischemic stroke in pigs. Plasma concentration (pg/ml) of **(A)** tumor necrosis factor alpha (TNF-α) and **(B)** interleukin-6 (IL-6) were measured prestroke (PRE, *n* = 7), 4 h poststroke (4 hrPS, *n* = 7 and 6, respectively), 12 h poststroke (12 hrPS, *n* = 7), 1 day poststroke (1DPS, *n* = 7), and 5 days poststroke (5DPS, *n* = 5). *P-*value: paired *t-*test comparing the mean prestroke values vs. each time point poststroke.

Elevated systemic inflammation has been associated with gut microbiome dysbiosis and correlated with increased brain infarction (McColl et al., 2007, 2008). Elevated systemic inflammation results in poor clinical outcomes and increased mortality in stroke patients (Di Napoli et al., 2001; Elkind et al., 2004), making it a key biomarker in stroke (Di Napoli and Papa, 2006). In MCAO pigs, plasma TNF-α was increased ∼28% at 4 h poststroke relative to prestroke levels (79.75 ± 6.00 pg/ml vs. 61.62 ± 6.38 pg/ml, respectively, *P* = 0.003, Figure 2A). Comparatively, TNF-α levels rapidly dropped following this peak and reached the lowest level at 1 day poststroke (43.75 ± 4.38 pg/ml, *P* = 0.003). TNF-α levels returned to prestroke levels by 5 days poststroke (53.36 ± 5.57 pg/ml). Similar to TNF-α, plasma IL-6 levels were significantly increased ∼20% at 12 h poststroke compared to prestroke (52.46 ± 2.44 pg/ml vs. 44.01 ± 0.85 pg/ml, respectively, *P* = 0.01) and returned to prestroke levels by 5 days poststroke (44.94 ± 1.07 pg/ml, Figure 2B), confirming an elevated inflammatory response during the acute stage of stroke in the pig model.

### Diversity of Fecal Microbiota Was Altered During the Acute Stage of Ischemic Stroke

Changes in diversity of gut microbiome are often an indicator of dysbiosis associated with disease pathology (Kriss et al., 2018). In MCAO pigs, microbial diversity and evenness were altered during the acute stage of stroke as shown in Table 1. The Shannon and evenness indices were reduced (*P* ≤ 0.05) at 1 day poststroke compared to prestroke but returned to prestroke levels at 3 days poststroke. However, the number of observed OTUs, which is an estimator of microbial richness, was not significantly affected (*P* ≥ 0.21) during the course of the study. Consequently, poststroke values were not significantly different from prestroke values (Table 1).

**TABLE 1:** Alpha-diversity was changed during the acute stage of ischemic stroke in a pig model.

Alpha-diversity indices		Prestroke (*n* = 7)	Poststroke		
			1 day (*n* = 7)	3 days (*n* = 6)	5 days (*n* = 4)
Number of observed OTUs	Mean	7,110	5,824	6,140	5,744
	SEM	1,130	407	222	344
	*P-*value	–	NS	NS	NS
Shannon	Mean	8.14	7.46	7.92	7.93
	SEM	0.40	0.27	0.18	0.31
	*P-*value	–	0.05	NS	NS
Evenness	Mean	0.638	0.596	0.630	0.635
	SEM	0.022	0.017	0.013	0.022
	*P-*value	–	0.02	NS	NS

*P-value, paired t-test comparing the mean prestroke values vs. each time point poststroke. SEM, standard error of mean; NS, not significant, P > 0.05.*

**FIGURE 3:**
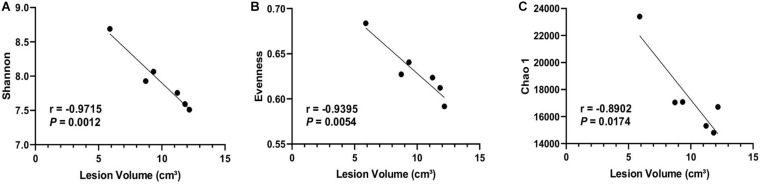
Microbial diversity was negatively correlated with brain lesion volume. Brain lesion volume measured 1 day poststroke was negatively correlated with alpha-diversity indices including **(A)** Shannon, **(B)** Evenness, and **(C)** Chao1 at 3 days poststroke (*n* = 6). Regression analysis was performed to evaluate associations between microbial composition and stroke severity. Pearson correlation coefficient and *P-*values are shown.

To further investigate the association between the dysbiosis and stroke severity, the correlations between microbial diversity and MRI results were assessed. The correlative analysis indicated that Shannon (*r* = −0.9715, *P* = 0.0012), Evenness (*r* = −0.9395, *P* = 0.0054), and Chao 1 (*r* = −0.8902, *P* = 0.0174) at day 3 poststroke was negatively associated with lesion volume measured by a high resolution of MRI (Figure 3), suggesting the lower microbial diversity poststroke was related to increased stroke severity at the acute stage of stroke.

**FIGURE 4:**
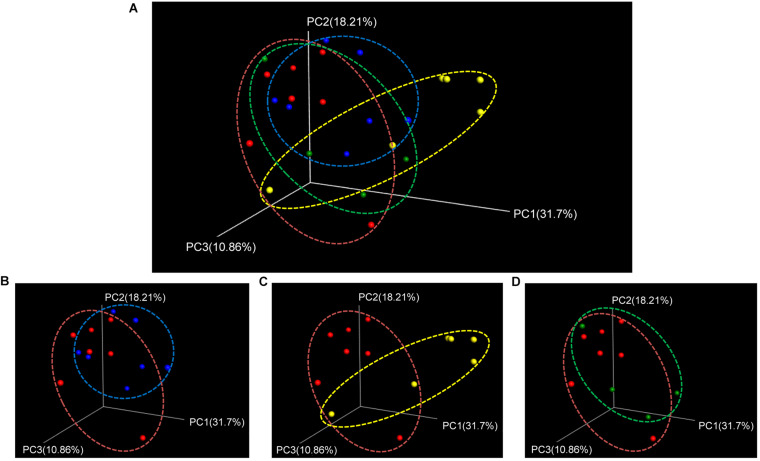
Beta-diversity (weighted UniFrac PCoA plots) showed trending differences between pre- and poststroke in middle cerebral artery occlusion (MCAO) pig model. **(A)** Beta-diversity changes during the acute stage of stroke were shown by PCoA plots. Different bacterial communities were compared between **(B)** prestroke (*n* = 7) vs. 1 day poststroke (*n* = 7), **(C)** prestroke vs. 3 days poststroke (*n* = 6), and **(D)** prestroke vs. 5 days poststroke (*n* = 4). Trending differences were observed between prestroke and 3 days poststroke (non-parametric *P-*values generated by the Bonferroni’s multiple comparisons test were used for inferences).

Beta-diversity was assessed using the weighted UniFrac distance matrix to investigate the similarity of microbial patterns among groups. There was a trend (*P* = 0.07) for overall differences across all time points (Figure 4). The most distinct separation in the UniFrac distance was observed between 3 days post- and prestroke (Figure 4C). Taken together, the alpha-diversity was decreased at 1 day poststroke, and beta-diversity was most distinctly different 3 days poststroke compared to prestroke.

### Stroke Altered Fecal Microbiome Composition

**FIGURE 5:**
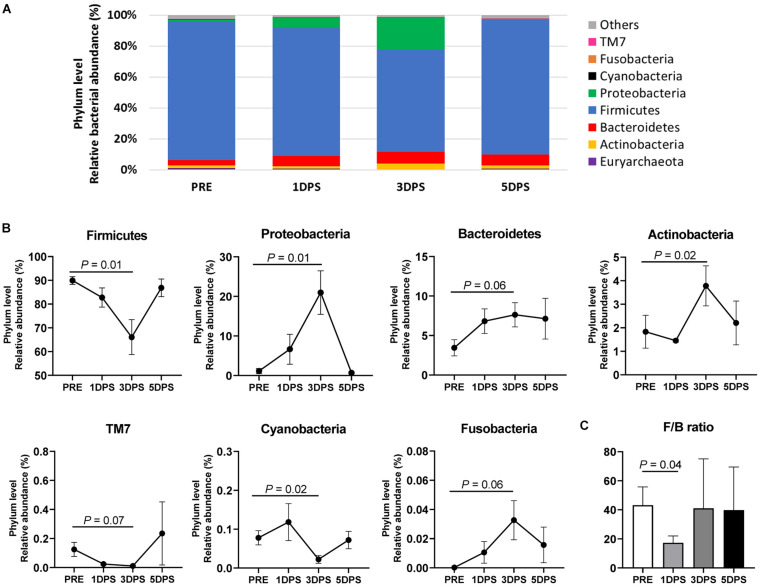
Stroke resulted in phylum level perturbations in gut microbiota between pre- and poststroke in MCAO pig model. **(A)** Mean relative abundances of bacterial phylum prestroke (PRE, *n* = 7), 1 day poststroke (1 DPS, *n* = 7), 3 days poststroke (3 DPS, *n* = 6), and 5 days poststroke (5 DPS, *n* = 4) are shown. **(B)** Bacterial phyla showed changes in the abundance (*P* < 0.10) during acute stage of stroke. **(C)** The ratio of Firmicutes to Bacteroidetes (F/B) at 1 day poststroke (1 DPS) was lower than that at prestroke (PRE), 3 days poststroke (3 DPS), and 5 days poststroke (5 DPS). *P*-value: paired *t*-test comparing the mean prestroke values vs. each time point poststroke.

**FIGURE 6:**
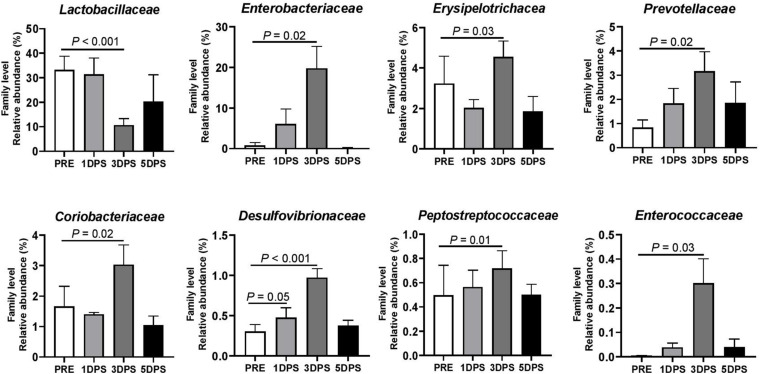
Stroke resulted in family level perturbations in gut microbiota between pre- and poststroke in MCAO pig model. Bacterial families showed changes in abundance (*P* ≤ 0.05) during the acute stage of stroke. *P*-value: Paired *t-*test comparing the mean prestroke values vs. each time point poststroke. Prestroke (PRE, *n* = 7), 1 day poststroke (1 DPS, *n* = 7), 3DPS (*n* = 6), and 5 DPS (*n* = 4).

**FIGURE 7:**
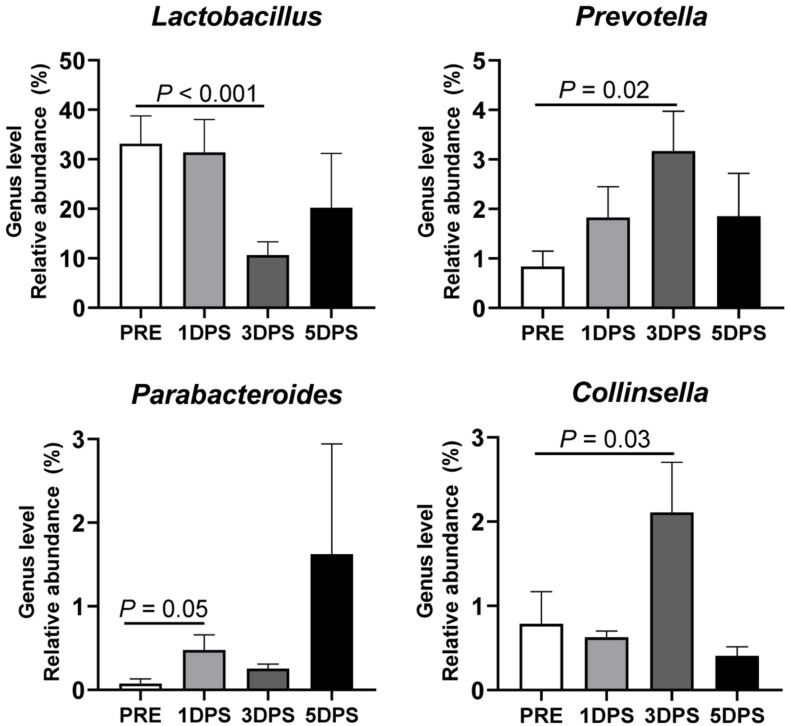
Stroke resulted in genus-level perturbations in gut microbiota between pre- and poststroke in middle cerebral artery occlusion (MCAO) pig model. The abundance of *Lactobacillus*, *Prevotella*, *Parabacteroides*, and *Collinsella* showed >1% relative abundance and demonstrated a significant change in abundance during acute stroke. *P-*value: Paired *t-*test comparing the mean prestroke values vs. each time point poststroke. Prestroke (PRE, *n* = 7), 1 day poststroke (1DPS, *n* = 7), 3 DPS (*n* = 6), and 5DPS (*n* = 4).

The composition changes in gut microbiota were evaluated at different taxonomic levels (phylum, family, and genus) during the acute stage of ischemic stroke (Figures 5–7). The most prevalent phyla prestroke were Firmicutes (89.94 ± 1.65%), followed by Bacteroidetes (3.45 ± 1.02%), Actinobacteria (1.83 ± 0.70%), and Proteobacteria (1.13 ± 0.65%, Figure 5). The composition of these four major phyla changed during the acute stage of stroke. At 3 days poststroke, the abundance of Firmicutes was decreased by 27% (66.08 ± 7.35% vs. 89.94 ± 1.65%, *P* = 0.01), while Proteobacteria significantly increased 19-fold relative to prestroke levels (20.96 ± 5.50% vs. 1.13 ± 0.65%, *P* = 0.01). At 5 days poststroke, both phyla returned to prestroke levels (Firmicutes, 86.86 ± 3.70% and Proteobacteria, 0.68 ± 0.19%). Similar to Proteobacteria, Actinobacteria reached their highest abundance 3 days poststroke (3.78 ± 0.85% vs. 1.83 ± 0.70%, *P* = 0.02 compared to prestroke) and showed comparable levels to prestroke at 5 days poststroke (2.20 ± 0.93%). The second most abundant phylum at prestroke, Bacteroidetes, tended to increase 3 days poststroke (7.63 ± 1.53% vs. 3.45 ± 1.02%, *P* = 0.06) compared to prestroke and remained consistent at 5 days poststroke (7.13 ± 2.58%, Figure 5B). The ratio of Firmicutes to Bacteroidetes was decreased ∼60% at 1 day poststroke compared to that of prestroke (17.33 ± 4.69% vs. 43.18 ± 12.59%, *P* = 0.04, Figure 5C) and returned to the levels observed prestroke at 3 days poststroke and remained stable, suggesting a significant microbial shift occurred at the acute stage of stroke. Other significant changes in bacterial phyla were observed in bacteria with relatively low abundance, including TM7, Cyanobacteria, and Fusobacteria, yet their abundance remained below 0.15% during the entire study (Figure 5B).

Consistent with phyla changes, a significant change in abundance was observed at the family level 3 days poststroke (Figure 6). The most abundant family prestroke was *Lactobacillaceae*, making up 33.13 ± 5.66% of the population. However, the population rapidly dropped to 10.63 ± 2.67% 3 days poststroke (*P* < 0.001) and increased to 20.19 ± 10.98% 5 days poststroke. The abundance of *Enterobacteriaceae*, *Erysipelotrichaceae*, *Prevotellaceae*, *Coriobacteriaceae*, *Desulfovibrionaceae*, *Peptostreptococcaceae*, and *Enterococcaceae* were increased up to 3 days poststroke and returned to prestroke levels at 5 days poststroke (Figure 6). Supplementary Figures 1, 2 show changes in the gut microbiome detected at the class and order levels, respectively.

Four bacterial genera were identified with relatively high abundance (>1%): *Lactobacillus*, *Prevotella*, *Parabacteroides*, and *Collinsella* (Figure 7). *Lactobacillus* had the greatest average abundance prestroke (33.13 ± 5.66%), and its presence reached the lowest point (10.63 ± 2.67%, *P* < 0.001) 3 days poststroke; however, it tended to return to prestroke levels at 5 days poststroke (20.19 ± 10.98%). Contrary to what was observed for *Lactobacillus*, the abundance of *Collinsella* (2.11 ± 0.59% vs. 0.79 ± 0.38%) and *Prevotella* (3.17 ± 0.81% vs. 0.84 ± 0.31%) were increased three to four times 3 days poststroke compared to prestroke levels (*P* ≤ 0.03). *Parabacteroides* was significantly increased at 1 day poststroke (0.48 ± 0.18%) compared to prestroke (0.08 ± 0.05%). Supplementary Figure 3 shows the 18 bacterial genera that were significantly altered during the acute stage of stroke in MCAO pigs. Stroke altered microbiota composition in MCAO pigs, with the majority of changes occurring 3 days poststroke as observed at the phylum, family, and genus levels.

Regression analysis conducted to better understand the changes in microbial composition between prestroke and 3 days poststroke and stroke severity at the acute stage of stroke indicates that changes in abundance of phylum Bacteroidetes, Proteobacteria, and Fusobacteria were positively correlated with lesion volume, MLS, and hemorrhage volume, while Firmicutes was negatively correlated with the stroke severity (Table 2). Moreover, changes in abundance of *Lactobacillaceae* and *Lactobacillus* were negatively related to the stroke severity (Table 2), supporting that the changes in microbial composition at the acute stage of stroke is closely related to stroke severity.

**TABLE 2:** Changes in gut microbiota composition for 3 days poststroke were correlated with lesion volume, midline shift, and hemorrhage volume in ischemic stroke in a pig model (*n* = 6).

		Lesion volume	MLS	Hemorrhage volume
Phylum	Bacteroidetes	*r* = 0.6318	*r* = 0.5920	*r* = 0.6310
		*P* = 0.0205	*P* = 0.0330	*P* = 0.0207
	Firmicutes	*r* = −0.7999	*r* = −0.7415	*r* = −0.8202
		*P* = 0.0010	*P* = 0.0037	*P* = 0.0006
	Proteobacteria	*r* = 0.8500	*r* = 0.7837	*r* = 0.8856
		*P* = 0.0002	*P* = 0.0015	*P* < 0.0001
	Fusobacteria	*r* = 0.6817	*r* = 0.5170	*r* = 0.4576
		*P* = 0.0103	NS	NS
Family	*Lactobacillaceae*	*r* = −0.7057	*r* = −0.7150	*r* = −0.6911
		*P* = 0.0070	*P* = 0.0060	*P* = 0.0089
	*Enterobacteriaceae*	*r* = 0.8440	*r* = 0.7715	*r* = 0.8802
		*P* = 0.0003	*P* = 0.0020	*P* < 0.0001
	*Prevotellaceae*	*r* = 0.7355	*r* = 0.7120	*r* = 0.7905
		*P* = 0.0042	*P* = 0.0063	*P* = 0.0013
	*Desulfovibrionaceae*	*r* = 0.7141	*r* = 0.7966	*r* = 0.7107
		*P* = 0.0061	*P* = 0.0011	*P* = 0.0065
	*Enterococcaceae*	*r* = 0.8270	*r* = 0.7174	*r* = 0.8710
		*P* = 0.0005	*P* = 0.0058	*P* = 0.0001
Genus	*Lactobacillus*	*r* = −0.7056	*r* = −0.7150	*r* = −0.6910
		*P* = 0.0071	*P* = 0.0060	*P* = 0.0089
	*Prevotella*	*r* = 0.7355	*r* = 0.7120	*r* = 0.7904
		*P* = 0.0042	*P* = 0.0063	*P* = 0.0013
	*Parabacteroides*	*r* = 0.6110	*r* = 0.5803	*r* = 0.5573
		*P* = 0.0265	*P* = 0.0376	*P* = 0.0479

*r, Pearson correlation coefficient; NS, not significant, P > 0.05.*

## Discussion

In this study, we investigated alterations in microbial composition during the acute stroke phase in a MCAO pig ischemic stroke model. The fecal microbiome was dynamically changed during this stage, as follows: (1) stroke reduced species diversity and evenness 1 day poststroke and changed bacterial community patterns (beta-diversity) among groups 3 days poststroke; (2) the ratio of Firmicutes to Bacteroidetes was decreased 1 day poststroke; (3) high abundance of Proteobacteria and low abundance of the genus *Lactobacillus* were observed 3 days poststroke. These results showed a dynamic compositional change in bacteria following a stroke event, particularly 3 days poststroke, and that this was transient with most microbiome metrics returning to prestroke levels by 5 days poststroke. Interestingly, increases in the systemic inflammatory response measured by circulating TNF-α and IL-6 were observed along with changes in fecal microbiome at the acute stage of stroke. This initial study demonstrates the plasticity of the gut microbiome during the acute stage of stroke, which occurred concurrently with systemic inflammation.

Microbial dysbiosis of the gastrointestinal tract has been reported in a number of neurological injuries and diseases including stroke (Swidsinski et al., 2012; Yin et al., 2015; Benakis et al., 2016; Singh et al., 2016; Prehn-Kristensen et al., 2018; Nicholson et al., 2019). These compositional changes in the gut microflora can be assessed by using alpha- and beta-diversity indices (Wagner et al., 2018). Alpha-diversity, representing the richness and diversity of a community, has been shown to decrease in attention-deficit/hyperactivity disorder (Prehn-Kristensen et al., 2018), autism spectrum (Ma et al., 2019) as well as brain injury (Singh et al., 2016; Nicholson et al., 2019). In accordance with the previous studies, our results showed the rapid reduction in alpha-diversity (Shannon index and evenness) responding to MCAO-induced stroke. Likewise, the differences in beta-diversity, representing overall similarity of bacterial communities, have been reported in stroke (Yin et al., 2015; Singh et al., 2016) and brain injury models (Prehn-Kristensen et al., 2018; Nicholson et al., 2019). Consistent with the shifts shown in brain injury rodent models (Nicholson et al., 2019), our beta-diversity analysis revealed the greatest difference at 3 days poststroke compared to prestroke. By 5 days poststroke, the distinctive microbiome pattern overlapped with the prestroke pattern, suggesting that beta-diversity recovered during the acute stage of stroke, although the microflora profile is not identical to prestroke. Overall, our findings demonstrate a reduced species diversity and evenness and trending changes in beta-diversity between pre- and poststroke, indicating that stroke alters the gut microbiome during the acute stage.

Firmicutes and Bacteroidetes are the two predominant phyla in human gut bacteria, and the ratios between these phyla are often used as a marker of dysbiosis or is seen as indicative of energy availability in the lower gastrointestinal tract (Eckburg et al., 2005). A decrease in the ratio of Firmicutes to Bacteroidetes (F/B ratio) has been reported in neurological (Rowin et al., 2017; Vogt et al., 2017; Nicholson et al., 2019) and inflammatory bowel diseases (Kabeerdoss et al., 2015). Stroked pigs in this study also showed significantly reduced F/B ratio 1 day poststroke. Previous obesity studies showed increases in F/B ratio (Ley et al., 2006; Koliada et al., 2017), which suggested that obese individuals that had a high abundance of Firmicutes may be more efficient at extracting energy from the diet in the form of volatile fatty acids produced in the lower gastrointestinal tract microbial fermentation (Turnbaugh et al., 2006; Munukka et al., 2012). The reduction in the F/B ratio observed in the current study suggests that stroke pigs may not be able to produce as many volatile fatty acids following stroke. Consequently, the role of the F/B ratio in a disease pathology needs to be further investigated (Liang et al., 2018).

Proteobacteria is a well-known phyla containing opportunistic pathogenic bacteria such as *Escherichia*, *Salmonella*, *Helicobacter*, and others (Rizzatti et al., 2017), and increased abundances have been observed in type 2 diabetes (Lambeth et al., 2015), obesity (Fei and Zhao, 2012), inflammatory bowel disease (Morgan et al., 2012), and neurological conditions including stroke (Yin et al., 2015; Nicholson et al., 2019). A rapid increase in Proteobacteria was observed 3 days poststroke in the current study, indicating an increase in the phyla following stroke onset. Stroked pigs had a greater abundance of *Enterobacteriaceae* and *Desulfovibrionaceae* 3 days poststroke. Enriched levels of the families *Enterobacteriaceae* and *Desulfovibrionaceae* were previously found in patients with a high risk of stroke (Zeng et al., 2019), and higher level of *Desulfovibrionaceae* was detected in patients following stroke (Yin et al., 2015). The increased abundance of Proteobacteria at the acute stage of stroke may play an important role in the development of systemic inflammation in stroke, potentially leading to more deleterious outcomes.

Lactic acid bacteria (LAB) may help in reducing inflammation and in controlling pathogen populations through the production of lactic acids. In addition, LAB produce important gut-derived metabolites such as short-chain fatty acids (George et al., 2018), which act as signaling molecules in immune responses (Rizzetto et al., 2018). Talani et al. (2020) demonstrates that implementation of gut *Bifidobacteria* improved cognitive behavior and hippocampal plasticity with increases in hippocampal BDNF in rats, suggesting the probiotics as a potential therapeutic treatment in brain diseases associated with cognitive functions. *Lactobacillus* is a common component of commercial human and animal probiotics (Salvetti et al., 2012). Low populations of *Lactobacillus* were found in patients with irritable bowel syndrome, HIV, type 1 diabetes, and multiple sclerosis (Heeney et al., 2018). A decrease in relative abundance of *Lactobacillus* was also observed in the present study 3 days poststroke. Contrary to our findings, Zeng et al. (2019) found enrichment of LAB in high-risk stroke patients and suggested that the presence of LAB in the gastrointestinal tract compensates for the loss of butyrate-producing bacteria in these individuals. Further research is needed to reconcile the discrepancies in the field. The results from this study suggest that the use of probiotics such as *Lactobacillus* at the acute stage may benefit stroke patients.

Increased populations of specific microflora may result in an increase in end products that are risk factors for stroke such as trimethylamine-N-oxide (TMAO). Trimethylamine, a precursor of TMAO, is produced by gut microbiota from dietary choline and is further metabolized to TMAO in the liver. Circulating TMAO has been reported to increase the buildup of atherosclerotic plaques in coronary vasculature, increasing the risks of stroke (Bennett et al., 2013; Tang et al., 2013). Specifically, the abundance of *Peptostreptococcaceae* and *Prevotella* were positively associated with circulating TMAO (Koeth et al., 2013). In the present study, pigs with stroke had increased abundances of both *Peptostreptococcaceae* and *Prevotella*, suggesting that the dysbiosis during the acute stage of stroke is potentially related to increased TMAO production. Additionally, a dysregulation of lipid profiles is considered as another risk factor for stroke. In stroke pigs, an increased abundance of *Coriobacteriaceae* was found, which was negatively correlated with blood triglycerides and low-density lipoprotein cholesterol in hyperlipidemia patients (Liu et al., 2018). The increase in *Coriobacteriaceae* observed in this study may be the result of compensatory mechanisms in response to stroke-induced changes in gastrointestinal conditions that alter the microbial ecology throughout the gut. Understanding the role of microflora on the regulation of metabolites associated with stroke may provide an insight on the development of novel therapeutic targets, as reviewed by Tonomura et al. (2020) indicating the role of bacterial metabolites such as TAMO and short-chain fatty acids in stroke.

Evidence on the interaction between the gut microbiota and stroke outcome have been cumulatively reported in humans and animal models. In humans, stroke dysbiosis was closely linked to severe stroke and unfavorable outcome (Yin et al., 2015; Xia et al., 2019). In mice model of stroke, disturbance of the gut microbiota increased intestinal proinflammatory T cells and have aggravated ischemic brain lesions (Benakis et al., 2016). In the current study, the changes in microbial diversity and microbiota composition were associated with stroke severity measured by high-resolution structural MRI at the acute stage of stroke. The lesion volume was negatively related with the alpha-diversity indexes suggesting that reduced microbial richness and evenness in stroke pigs are related to the high severity of stroke. Moreover, we found that potential pathogenic bacteria Proteobacteria, *Enterobacteriaceae*, and *Desulfovibrionaceae* were increased and beneficial bacteria *Lactobacillaceae* and *Lactobacillus* were decreased 3 days poststroke. Interestingly, abundance of the pathogenic bacteria was positively related to the lesion volume, MLS, and hemorrhage volume, while that of beneficial bacteria was negatively related to the stroke severity, proposing that gut dysbiosis may be a potential indicator to identify prognostic and therapeutic target for stroke.

The role of inflammation in neurological diseases has been widely recognized (Di Napoli et al., 2001; Audebert Heinrich et al., 2004; Elkind et al., 2004; McColl et al., 2007, 2008), and alterations in the bacterial community due to disease have been correlated with changes in inflammatory responses (Benakis et al., 2016; Winek et al., 2016; Yamashiro et al., 2017; Spychala et al., 2018; Zeraati et al., 2019). Consistent with previous findings, increased plasma levels of proinflammatory cytokines were observed in the current study, concurrent with the changes in gut microbiome composition. Increased gut permeability and translocation of bacteria to host tissues have been previously reported in stroke conditions (Crapser et al., 2016; Stanley et al., 2016), supporting the involvement of the gut microbial population in stroke pathophysiology. It is well known that lipopolysaccharides (LPS) from the cell wall of Gram-negative bacteria (Hurley, 1995) trigger an immune response by binding to Toll-like receptor 4 in endothelial cells, activating monocytes/macrophages, and nuclear factor kappa B signaling cascades, resulting in the production of proinflammatory cytokines such as TNF-α (Cario et al., 2002; Mafra et al., 2014). Proinflammatory cytokines contribute to the disruption of tight junction proteins between the epithelial cells, leading to an increase in gut permeability (Katzenberger et al., 2015) as well as translocation of bacteria and microbial-derived end products into the blood stream in what is known as “leaky gut syndrome.” Leaky gut syndrome results in a cycle of increasing inflammation that is detrimental to stroke patients, which could potentially be mitigated by therapeutic treatments that alter the composition of the gut microbial composition.

## Conclusion

The present study demonstrated, for the first time using a large translational animal model such as swine, the plasticity of the gut microbiome during the acute stage of stroke. These changes included significant shifts in microbial diversity, the ratio of Firmicutes to Bacteroidetes, and the abundance of Proteobacteria and *Lactobacillus*. Importantly, the microbial changes were significantly correlated with the severity of brain lesion measured by MRI. Given the significant degree of physiological similarities between swine and humans, findings from the current study contribute to increasing our understanding of the pathophysiology of stroke in human patients. Future studies investigating the role of the microbiome and its effect on the stroke immune response are warranted to understand the effect of therapeutic treatments on the gut microbiota in stroke patients.

## Data Availability Statement

The original contributions presented in the study are publicly available. This data can be found here: https://www.mg-rast.org, under accession number mgm4901524.3.

## Ethics Statement

The animal study and experimental procedures were reviewed and approved by the University of Georgia Institutional Animal Care and Use Committee and the study was conducted in accordance with the recommendations of the NIH’s guide for the Use and Care of Laboratory Animals (AUP approval number: A2017 07-019-Y2-A16).

## Author Contributions

JJ performed the experiments, collected the data, analyzed the data, and wrote the manuscript. JL analyzed the data and wrote the manuscript. EK and EW performed the experiments, collected the data, and administered the project. KS and XF performed the experiments and collected the data. HK administered the project. SP and MR managed the experimental methodology. TC conceptualized the project. FW contributed to funding acquisition, administered the project, and wrote the manuscript. HP conceptualized the project, contributed to funding acquisition, administered the project, and wrote the manuscript. All authors contributed to the article and approved the submitted version.

## Conflict of Interest

The authors declare that the research was conducted in the absence of any commercial or financial relationships that could be construed as a potential conflict of interest.

## Footnotes

1 https://dna.uga.edu/
