# Supplementary material for: Dynamic Changes in the Gut Microbiome at the Acute Stage of Ischemic Stroke in a Pig Model
Source: Front Neurosci. 2020 Dec 3;14:587986. doi: 10.3389/fnins.2020.587986 (PMC7744295; doi:10.3389/fnins.2020.587986)
Supplement: Supplementary file 1 [file Presentation_1.pdf]

Supplementary Material

Supplementary Material

Supplementary Figures

A

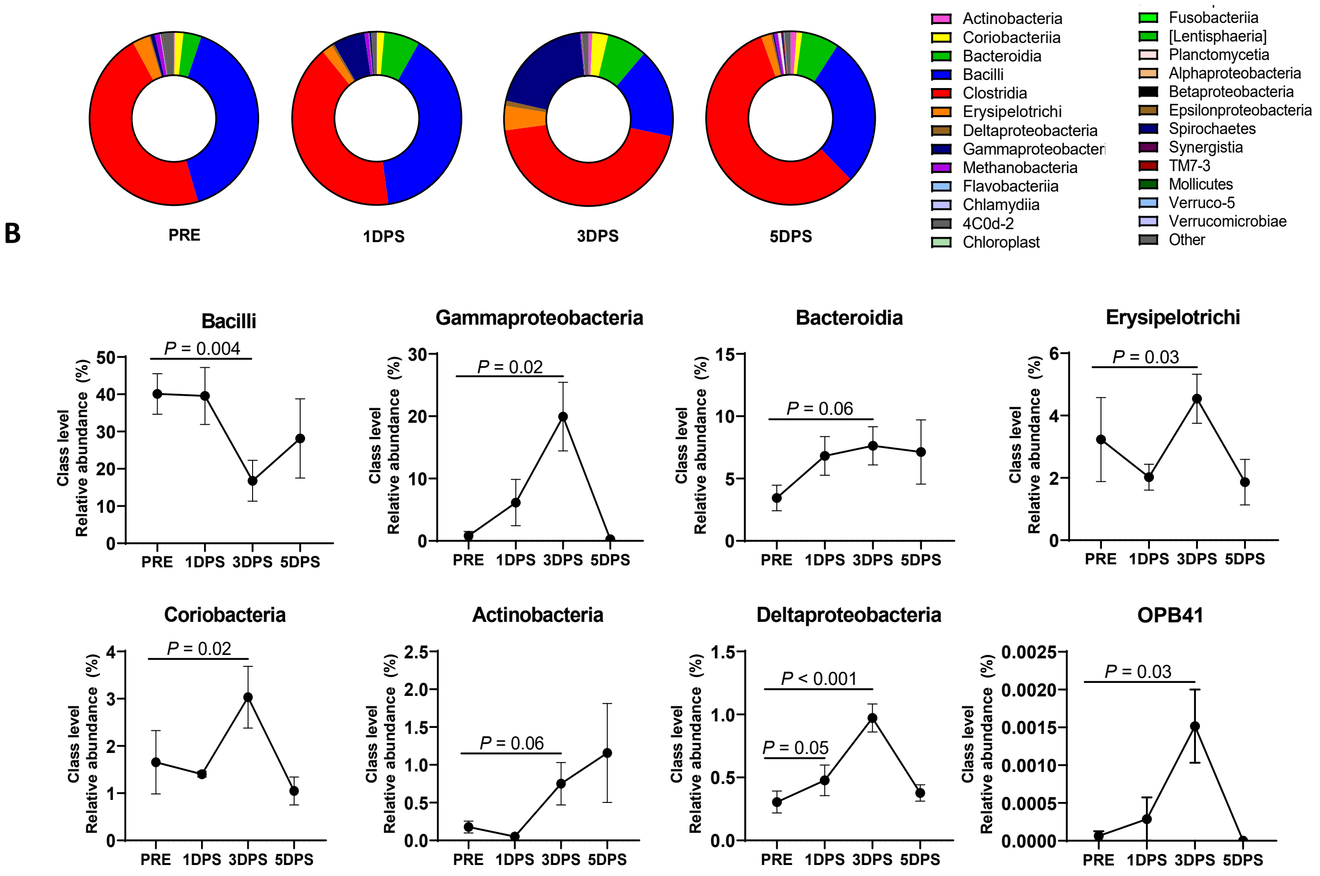

Supplementary Figure 1. Changes in gut microbiome were detected at the class level pre- and post-stroke. **(A)** Circle plots showed changes in mean relative abundance of all bacterial classes pre-stroke (PRE, n=7), 1 day post-stroke (1DPS, n=7), 3 days post-stroke (3DPS, n=6), and 5 days post-stroke (5DPS, n=4). **(B)** Bacterial classes showed changes in abundance ($P < 0.10$) during the acute stage of stroke. *P*-value: Paired t-test comparing the mean pre-stroke values vs. each time point post-stroke.

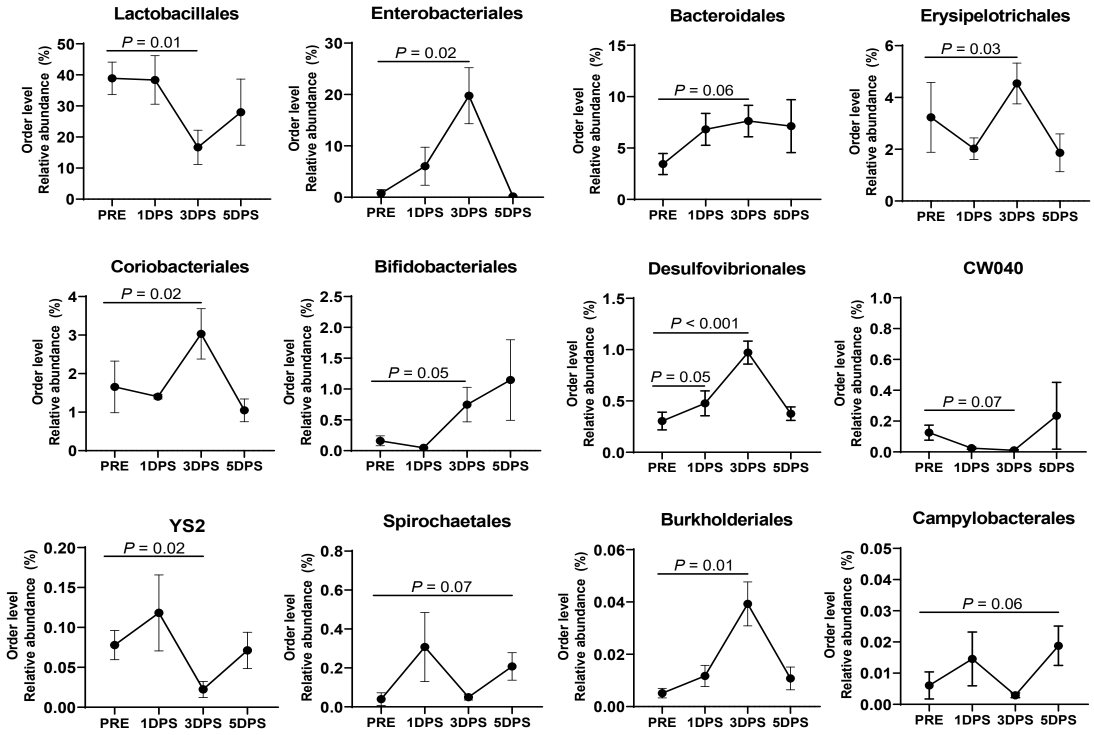

Supplementary Figure 2. Changes in gut microbiome were detected at the order level pre- and post-stroke. Bacterial orders showed changes in abundance ($P < 0.10$) during the acute stroke stage. P-value: Paired t-test comparing the mean pre-stroke values vs. each time point post-stroke. PRE (n=7), 1DPS (n=7), 3DPS (n=6), 5DPS (n=4).

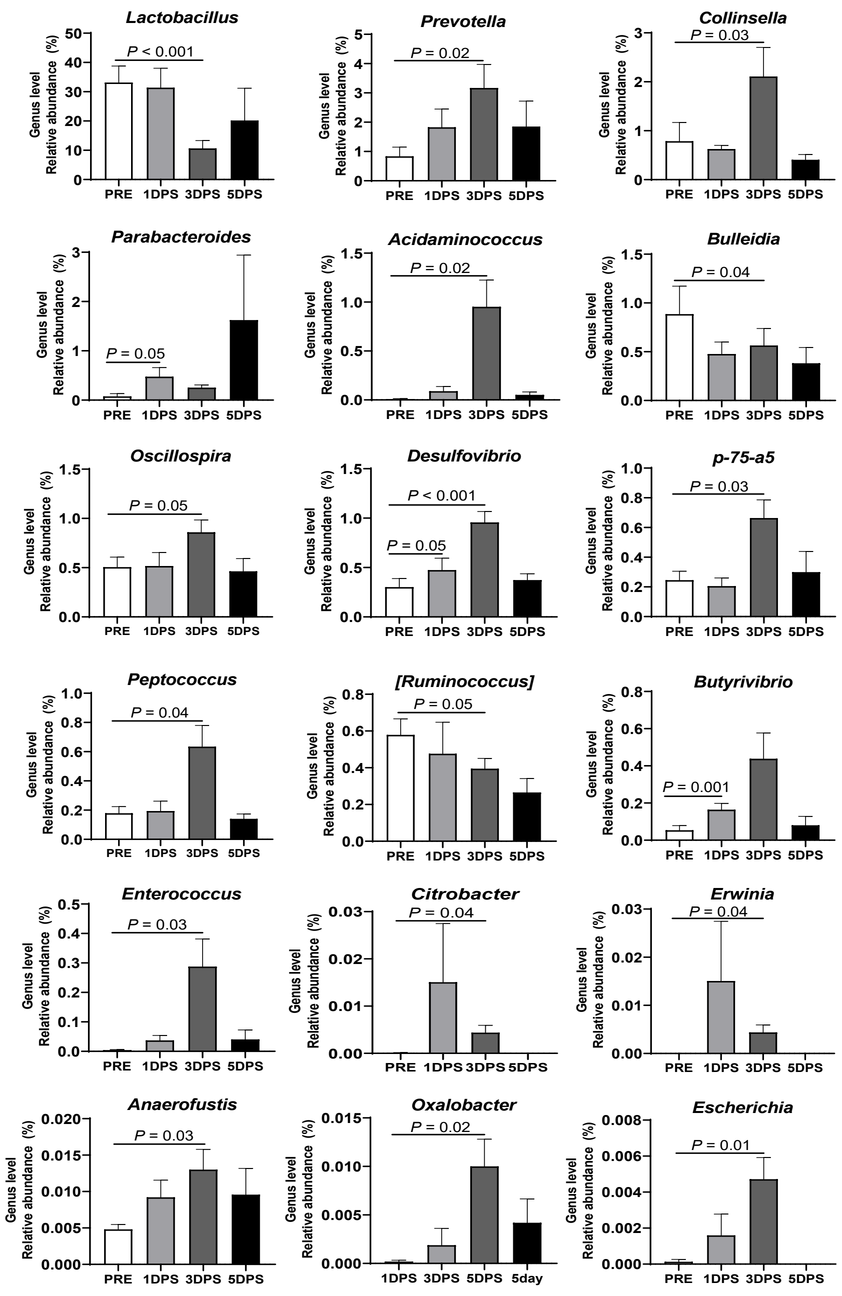

Supplementary Figure 3. Changes in gut microbiome were detected at the genus level pre- and post-stroke. Bacterial genus showed changes in abundance ($P \leq 0.05$) during the acute stroke stage. *P*-value: Paired t-test comparing the mean pre-stroke values vs. each time point post-stroke. PRE (n=7), 1DPS (n=7), 3DPS (n=6), 5DPS (n=4).
